# FPGA Programming Challenges When Estimating Power Spectral Density and Autocorrelation in Coherent Doppler Lidar Systems for Wind Sensing †

**DOI:** 10.3390/s25030973

**Published:** 2025-02-06

**Authors:** Sameh Abdelazim, David Santoro, Fred Moshary

**Affiliations:** 1 Gildar Haase School of Computer Sciences and Engineering, Fairleigh Dickinson University, 1000 River Rd., Teaneck, NJ 07666, USA; 2 The Remote Sensing Laboratory, The City College of New York, 140th St. and St. Nicolas Av., New York, NY 10031, USA

**Keywords:** doppler lidar, wind measurements, FPGA, remote sensing, coherent detection

## Abstract

In this paper, we present the logic designs of two FPGA hardware programming algorithms implemented for a Coherent Doppler Lidar system used in wind sensing. The first algorithm divides the received time-domain signals into segments, each corresponding to a specific spatial resolution. It then calculates the power spectrum for each segment and accumulates these spectra over 10,000 pulse returns. The second algorithm computes the autocorrelation of the received signals and accumulates the results over the same number of pulses. Both signal pre-processing algorithms are initially developed as logic designs and compiled using the Xilinx System Generator toolset to produce a hardware VLSI image. This image is subsequently programmed into an FPGA. However, the hardware implementation of these algorithms presents several challenges: (1) bit growth: multiplication operations in the binary number system significantly increase the number of bits, complicating hardware implementation. (2) Memory constraints: onboard RAM arrays of sufficient size are lacking for accumulating vectors of the calculated Fast Fourier Transforms (FFTs) or autocorrelations. (3) Signal drive issues: large fan-out in the logic design leads to significant capacitance, restricting the driving capabilities of transistor output signals. This article discusses the solutions devised to overcome these challenges. Additionally, it presents atmospheric wind measurements obtained using the two algorithms.

## 1. Introduction

Light Detection and Ranging (LIDAR) techniques are based on transmitting laser signals into the atmosphere then detecting and analyzing the backscattered signals that result from interactions with atmospheric aerosols and particles [1]. Coherent Doppler Lidar (CDL) systems are widely used in atmospheric measurements and remote sensing applications such as measuring atmospheric wind velocity, turbulence, aerosol concentration, atmospheric constituents and pollutants, and cloud heights. An early wind sensing was reported by Huffaker et al. [2] in 1970, where a continuous-wave (CW) 10.6-μm laser was used. In the 1980s, CDL systems with solid-state lasers were adopted for their reduced size and weight as well as their increased reliability and lifetime [3,4]. These solid-state lasers allowed for shorter wavelengths, which, in turn, allowed for higher spectral resolution. Significant advancements have been made in systems utilizing 2-μm pulsed lasers for wind measurements [3,5]. Systems with wavelengths less than 2-μm have been advancing the field. Karlsson et al. [6] introduced a 1.5-μm CW all-fiber CDL system for wind sensing.

At the Remote Sensing Laboratory (RSL) of the City College of New York (CCNY), an all-fiber CDL system for wind sensing has been developed, tested, and operated [7]. This system leverages a 1.5-μm fiber laser to capitalize on the availability and cost-effectiveness of telecommunication-grade optical components at this wavelength. The laser operates at a pulse frequency rate (PRF) of 20 kHz. Data acquisition is conducted at a sampling rate of 400 MHz using a 14-bit analog-to-digital converter (ADC) card equipped with a Virtex-5 FPGA (Innovative Integration, Camarillo, CA, USA).

The system’s high PFR and sampling rate result in the need to transmit 800 MB/s of backscattered signals to the host PC, along with the processing of 64 FFTs, each consisting of 128 sample vectors, with a 50-μs interval between consecutive laser pulses. The challenge extended beyond merely transmitting unprocessed signals. Even if such transmissions were possible, performing signal processing on the host PC is impossible. Without the FPGA, the host PC would be incapable of processing backscattered signals in real time. This limitation arises because the rate at which backscattered signals are received far exceeds the capacity of software-based processing.

To address this limitation, pre-processing algorithms were implemented directly as logic circuits on the FPGA. This design ensures that backscattered signals are processed in real time at the hardware level immediately after digitization, prior to being streamed to the host PC. FPGAs provide a significant computational advantage in signal processing compared to software-based applications [8,9]. FPGAs have been widely used in applications such as digital signal processing and deep learning, and in charge-coupled device (CCD) detectors [10]. Two signal processing algorithms are introduced in this paper. (1) A Fast Fourier Transform (FFT)-based algorithm: received signals are segmented based on the desired spatial resolution; an FFT is computed for each segment; and the square modulus of the complex FFT is calculated. These square moduli are accumulated over 10,000 pulses. (2) Autocorrelation-based algorithm: received signals are processed to extract in-phase (I) and quadrature (Q) components. The signals are filtered and down-sampled and an autocorrelation is computed. The results are accumulated over 10,000 pulses.

This paper details the implementation of these signal processing algorithms on the FPGA. Section 2 outlines the system configuration, while Section 3 describes the data acquisition process and signal processing algorithms. Section 4 focuses on the digital design of the FFT pre-processing algorithm, and Section 5 discusses the autocorrelation-based signal pre-processing algorithm. Finally, Section 6 presents the host PC signal processing procedures and reports wind measurement results.

## 2. Data Acquisition and Signal Processing Techniques

Digitizing received signals at 400 MSPS using a 14-bit ADC generates a data rate of 800 MB/s. Streaming such a large volume of data from the ADC to a host PC demands specialized hardware and consumes substantial CPU resources on the host system.

The challenge extended beyond merely transmitting unprocessed signals. Even if such transmissions were possible, performing signal processing on the host PC is impossible. Without the FPGA, the host PC would be incapable of processing backscattered signals in real time. This limitation arises because the rate at which backscattered signals are received far exceeds the capacity of software-based processing. In the absence of an FPGA, the signals would need to be streamed and processed offline, significantly increasing the overall processing time. Moreover, over the course of a single day, this system can produce over 67 TBs of data, making long-term data storage and archiving highly impractical.

**Figure 1:**
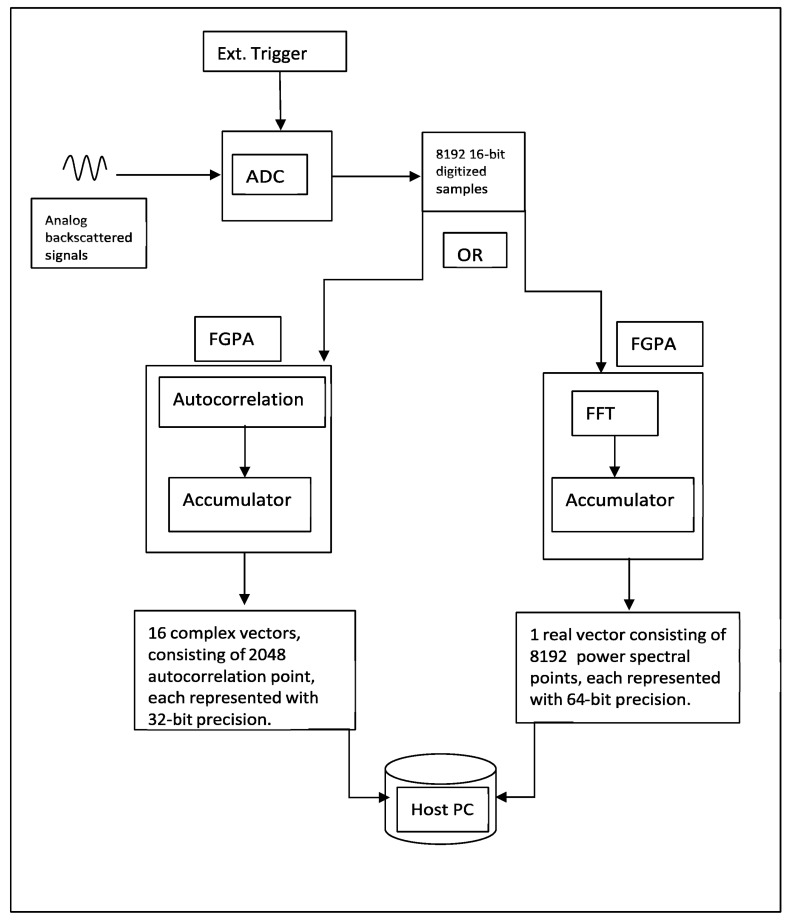
Data acquisition is synchronized with the transmission of each laser pulse using an external trigger. A total of 8191 time-domain samples are then processed using FFT or autocorrelation algorithms programed into an FPGA.

Signal acquisition is synchronized, with the transmission of each laser pulse using an external trigger, as shown in Figure 1. Each laser pulse, traveling back and forth within 50 microseconds (corresponding to a PFR of 20 kHz), generates 20,000 samples. We chose to process only 8192 samples, corresponding to approximately 3.1 km, because the signal’s power is extremely weak beyond this distance. These samples can be divided into 64 vectors, each containing 128 samples. The host PC would then need to compute 64 FFTs, each comprising 128 samples and corresponding to a range resolution of 48 m per gate, within the 50-μs time frame, while also handling additional post-processing tasks, data archiving, and visualization. The sheer volume of computations required in such a short interval exceeds the capabilities of software-based processing. Our experiments demonstrated that, under such conditions, only 10% of the received signals could be processed before new data became available. Consequently, this approach would result in a loss of 90% of the received signals, allowing for the processing of only a small fraction of the data.

Real-time signal processing for a 20 kHz PFR lidar system requires segmentation of the backscattered signal from each pulse—comprising 20,000 time-domain samples (at 400 MSPS)—into segments of specific lengths corresponding to the desired spatial resolutions. In the Fast Fourier Transform (FFT) algorithm, the power spectrum of each segment of the received signals is estimated by computing the squared magnitudes of its FFT. To meet the stringent timing requirements, the segmentation of signals from a single pulse and the FFT calculations for each segment must be completed within 50 μs, the interval between consecutive laser pulses. This processing speed cannot be achieved with software alone. Hence, an FPGA is programmed to handle the pre-processing of backscattered signals as they are acquired by the ADC.

The FPGA processes the data by calculating the power spectrum for the signals within each range gate and accumulating these spectra over 10,000 pulse returns. The result is a vector of 8192 frequency-domain points, each of 64 bits, representing periodograms of the accumulated signals over 0.5 s (10,000 pulses). This approach reduces the data transfer rate from the ADC to the host PC from 800 Mbytes/s to just 64 Kbytes/s, significantly optimizing system performance. The autocorrelation algorithm calculates an autocorrelation array of the received signals over a specified number of lags. In our design, 16 lags were carefully chosen [11]. The autocorrelation data are accumulated over 10,000 pulse returns, significantly reducing the data transfer rate from the ADC card to the host PC to approximately a few hundred kilobytes per second. This processing technique enables adjustable spatial resolution, giving it a distinct advantage over the FFT-based methods. To achieve this, the autocorrelation array is first computed for the entire vector of received signals, consisting of 8192 samples. A specific range distance is then selected by calculating the power spectrum of an autocorrelation vector, with its sample count corresponding to the desired range.

The autocorrelation array is derived by creating the in-phase (I) and quadrature (Q) components of the received signals. This is achieved by multiplying the received signals with cosine and sine waves oscillating at 84 MHz. It is worth noting that the CDL system uses a heterodyne detection where the outgoing pulse has been shifted by 84 MHz. Each multiplication generates high- and low-frequency components oscillating at the sum and difference of 84 MHz and the frequency of the received signals. A low-pass filter is applied to both I and Q components to remove the high-frequency components.

Subsequently, the signals undergo down-sampling by a factor of four. This is achieved by retaining one sample and discarding the next three. As a result, the original sampling time of 2.5 ns (400 MHz) is effectively reduced to 10 ns (100 MHz). The I and Q components together form a complex time sequence, expressed as follows:

*S_i_* = *I_i_* + *j Q_i_* (1)

where *S* is a complex time sample, *j* is $\sqrt{-1}$, *i* is the time sample index, and *I* and *Q* are in-phase and quadrature samples, respectively. An n×m autocorrelation array is generated by multiplying a complex time sequence with its delayed complex conjugate sequence. Here, n represents the total number of samples in the decimated I and Q vectors (2048), while m corresponds to the number of autocorrelation lags (16). This process is repeated over 10,000 laser pulses, during which the autocorrelation array is accumulated and subsequently streamed to the host PC. The design and implementation of the I–Q generator, low-pass filter, down-sampler, and autocorrelation array calculation circuit will be discussed in the following sections.

The signal pre-processing algorithm is implemented as a logic design, compiled using the Xilinx System Generator toolset (Xilinx, San Jose, CA, USA) to produce a hardware VLSI image. This image is programmed into an FPGA. Detailed descriptions of each pre-processing technique are provided in the subsequent sections.

## 3. FFT Pre-Processing Algorithm

In the FFT algorithm, the power spectra of segmented received signals are derived by computing the squared magnitudes of their respective FFTs, followed by the accumulation of these values over 10,000 pulse returns. The underlying logic design for this algorithm incorporates the following key logic circuits.

### 3.1. FFT Computing Digital Circuit

**Figure 2:**
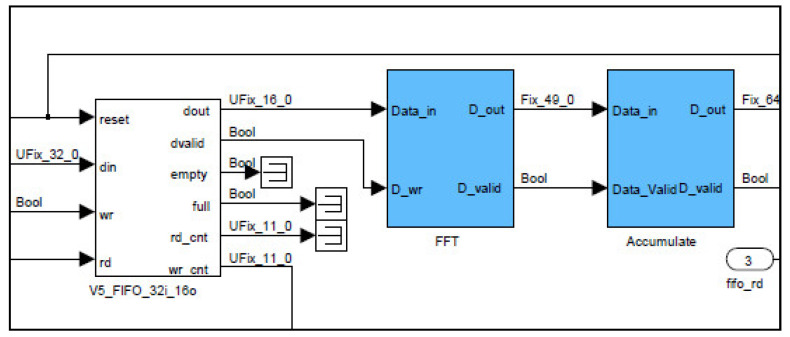
The digital circuit, as implemented on the FPGA to convert the combined 32-bit words into two separate 16-bit words, followed by calculating the FFT, and then accumulating the resulting power spectra.

FPGA logic circuits operate at a clock frequency of 250 MHz. Consequently, pairs of received signal samples are combined into 32-bit words to accommodate the ADC’s 400 MSPS data flow rate. Before any signal processing can occur, these 32-bit words must be decomposed back into their original 16-bit samples. This is achieved using a 32-bit to 16-bit converter circuit, as shown in Figure 2. Reducing the sample width from 32 bits to 16 bits effectively reduces the data flow rate, which could potentially lead to sample overflow and, consequently, data loss. To mitigate this issue, only 8192 samples are retained for processing at each rising edge of an external trigger, synchronized with each laser pulse, while the remaining samples are discarded. This approach ensures that the maximum measurable distance is approximately 3.1 km. However, signals from ranges beyond this threshold are weak and do not yield reliable results.

**Figure 3:**
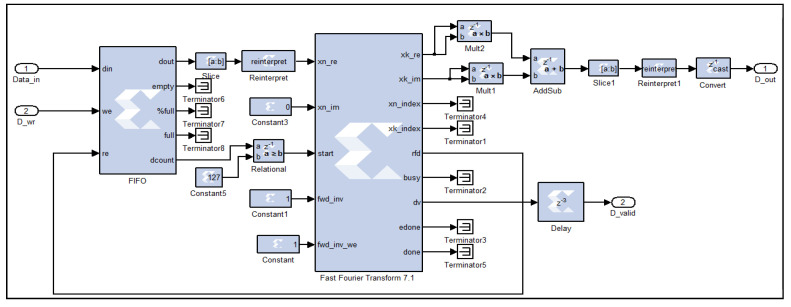
FFT computing digital circuit, as implemented on the FPGA.

To compute the FFT of signals acquired from range gates, each spanning 48 m, a logic circuit (Xilinx Fast Fourier Transform 7.1) initiates the calculation process once it has received a complete vector of 128 16-bit real samples, with the imaginary component set to zero, as the acquired time-domain signals are purely real. This is accomplished by monitoring the sample count from the preceding First In First Out (FIFO) buffer, and when the count reaches 128, a logic high signal triggers the FFT computation, as shown in Figure 3. The output samples are complex, increasing the data width to 25 bits. To calculate the square modulus of the FFT, the output is multiplied by its complex conjugate. This is achieved using two multiplication circuits—one to square the real part and the other to square the imaginary part of each power spectrum word. The squared real and imaginary components are subsequently summed in an addition circuit. It is important to note that the resulting square modulus words have a width of 50 bits, due to the squaring of 25-bit words and the subsequent addition operations.

### 3.2. Accumulator Digital Circuit

To accumulate the power spectra from all 64 range gates, each containing 128 words of 50 bits, over 10,000 pulse returns, an addressable memory array of 8192 × 64 bits is required. Accumulating a 50-bit word 10,000 times results in an increase in data width by up to 14 bits. This task can be efficiently achieved by storing the initial set of 10,000 vectors in an 8192 × 64-bit RAM, and subsequently accumulating the remaining vectors by updating the contents of each memory location with the sum of its current value and the newly arriving word. This process is repeated for all 10,000 vectors, after which the accumulated vector is passed on to the next processing stage.

However, the Xilinx Virtex 5 FPGA provides static random-access memory (SRAM) blocks that operate at a much lower frequency than that required to handle the incoming data rate. In contrast, it offers FIFO memory blocks that operate at the necessary frequency. As a result, a specialized logic architecture utilizing a FIFO memory block of size 8192 × 64 bits was designed to efficiently perform the accumulation of the square modulus of the power spectra.

In FIFO memory systems, data are stored and retrieved in a sequential manner, such that the first piece of data entered is the first one to be retrieved. As a result, direct access to specific memory locations is not possible. To access the content of a particular memory location, all preceding data in the stream must first be read. To facilitate the accumulation process in this environment, a specialized logic circuit is designed to function as a circular buffer. Initially, the power spectra vector from a single pulse is stored in a FIFO memory block via a multiplexer. Upon the arrival of a new vector from the next pulse return, the previously stored vector is read from the FIFO. This vector is then aligned with the incoming vector, and the two are summed. The resulting summed vector is written back into the FIFO, thus forming the accumulated power spectra from the two vectors.

**Figure 4:**
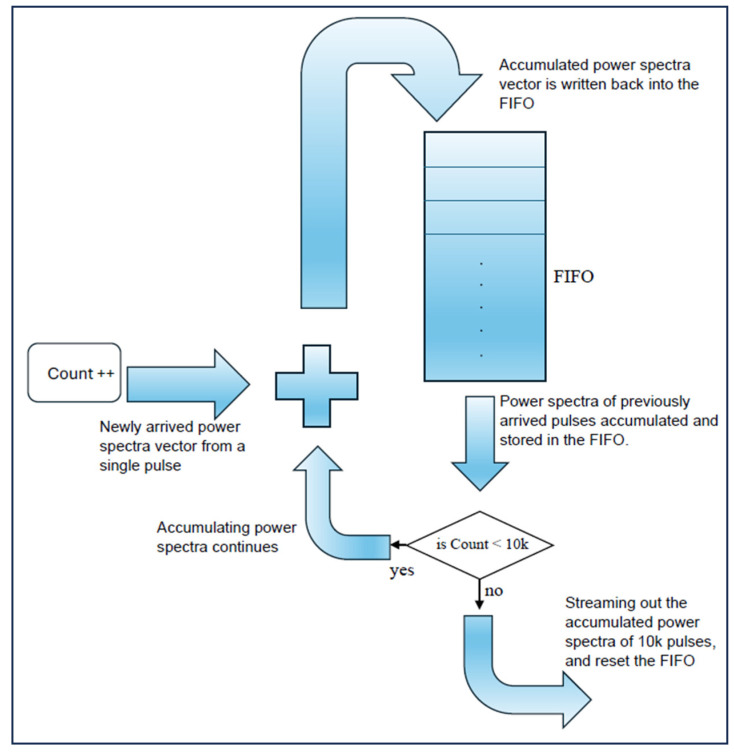
Power spectra accumulation technique using FIFO memory blocks instead of RAMs, as implemented on the FPGA.

As a result, while the new vector is entering the FIFO for summation, the previous vector begins to exit. This simultaneous reading and writing process occurs continuously. The accumulation process continues until a counter circuit reaches a value of 81,920,000, marking the conclusion of the accumulation of all words across the 10,000 power spectra vectors, as shown in Figure 4. A comparator circuit compares the counter’s value with this constant threshold, and based on the comparison, it controls a multiplexer circuit. The multiplexer selects between two inputs (either the summed power spectra words during accumulation or the zeros) to reset the FIFO contents once the 10,000 vectors have been processed.

Once the accumulation is complete, the accumulated power spectra vector is read from the FIFO and transferred to an output buffer for transmission to the host PC. Simultaneously, a vector of zeros is written to reset the FIFO contents. During this reset process, newly arriving vectors are ignored as the FIFO contents are streamed out and reset. Although a single power spectra vector is lost every 10,000 pulses, this loss has a negligible impact on the received signal power. A second comparator circuit governs the end of the FIFO reset cycle and the start of accumulating a new set of 10,000 power spectra vectors. This is achieved by comparing the counter’s total with the constant value of 81,936,384. Once the comparator’s output becomes true, the counter is reset, and the multiplexer begins outputting the summed power spectra words again, marking the beginning of a new accumulation cycle.

Arithmetic operations using hardware-based binary systems necessitate careful management of data width changes. For instance, multiplying two 16-bit numbers yields a 32-bit result, effectively increasing the data width. In signal processing, particularly in the Fast Fourier Transform (FFT) pre-processing stage, a 16-bit real input is expanded into a 25-bit complex output through the FFT logic. This 25-bit complex output is further extended to 50 bits during square modulus computations. Accumulating these 50-bit values over 10,000 iterations can cause the data width to increase further, potentially reaching 64 bits. Managing these expansions requires thoughtful design considerations, such as selecting appropriately sized buffers and ensuring accurate interpretation of the streamed output data.

## 4. FPGA Autocorrelation Pre-Processing

**Figure 5:**
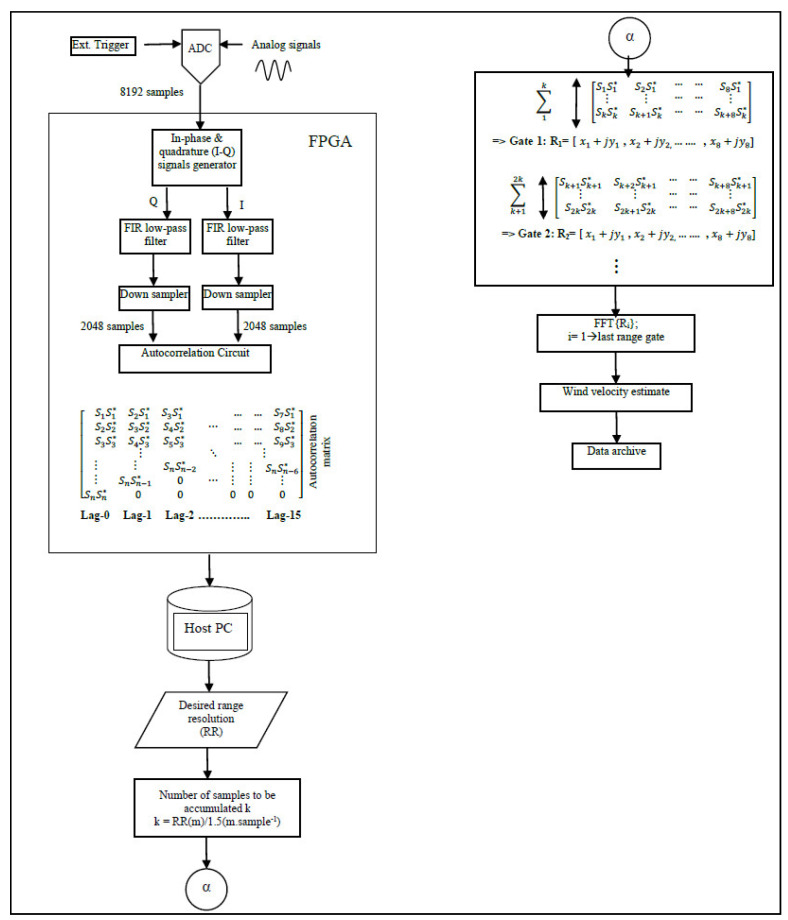
In the autocorrelation algorithm, in-phase and quadrature components are generated, passed through a low-pass filter, and down-sampled to build a 16-lag matrix. Processing of the autocorrelation matrix is conducted on the host PC, where a desired range gate can be chosen.

The autocorrelation algorithm encompasses several stages, including generating in-phase (I) and quadrature-phase (Q) signal components, filtering out high-frequency elements, down-sampling, and constructing an autocorrelation array. In heterodyne optical detection, local oscillator and backscattered signals are optically mixed through an optical coupler. The resulting mixed signal is then applied to a balanced photodetector. The power spectrum of received signals is found by calculating the FFT of the autocorrelation, as shown in the following equations:

(2) $$R(\tau )=\int_{-\infty }^{\infty }f(t)f(t+\tau )$$

(3) $$G(f)=\int_{-\infty }^{\infty }R(\tau )e^{-j2\pi f\tau }dt$$

where *f*(*t*) represents time-domain signals, *R*(*τ*) is the signals’ autocorrelation, *G*(*f*) is the Fourier Transform (power spectrum). In this technique, digitized received signals are split into two paths. One path is mixed with a cosine signal oscillating at 84 MHz to produce an in-phase (I) component, whereas the other path is mixed with a sinusoidal signal also oscillating at 84 MHz to produce a (Q) component [12]. One advantage of using this signal processing technique is the flexibility in choosing spatial resolution, as shown in Figure 5.

### 4.1. Digital Circuit Design for Generating I and Q Signals

**Figure 6:**
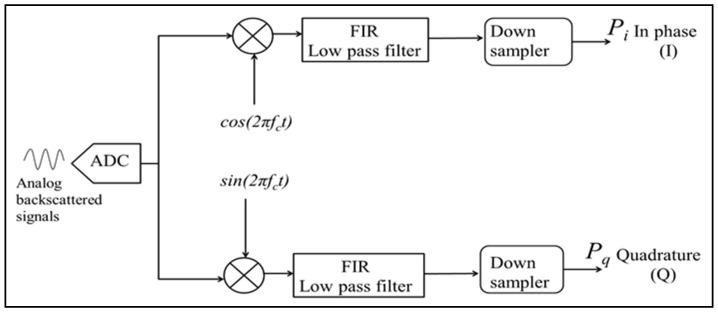
The autocorrelation algorithm is structured around a block diagram, where the input signals are processed through two distinct paths. In the first path, the signals are multiplied by a cosine function to generate the in-phase (I) component. Simultaneously, the second path involves multiplication by a sine function to produce the quadrature (Q) component. Both the I and Q signals undergo filtering to remove high-frequency components using finite impulse response (FIR) filters, and this is followed by a down-sampling process to prepare the data for subsequent stages of the algorithm.

To derive the in-phase (I) signals, backscattered input signals (8192 samples, each 16-bits wide) are multiplied by a cosine reference vector, whereas the quadrature signals are derived by multiplying the received signals by a sine reference vector, as shown in Figure 6.

**Figure 7:**
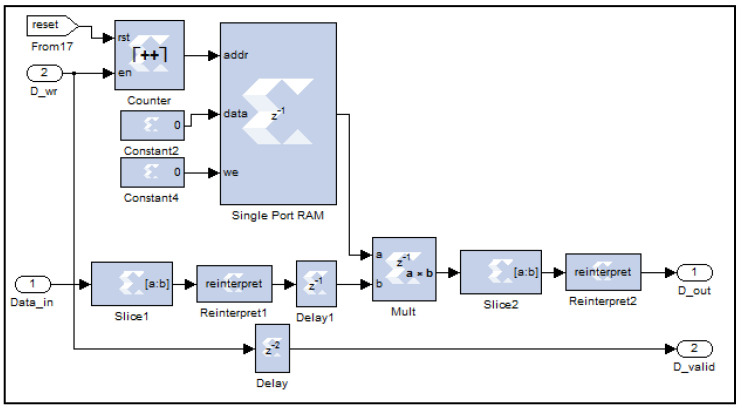
I and Q generation digital circuit.

This reference vector, stored in a RAM block, is an 8k vector of cos (2*πf_c_*), with each sample represented by a 3-bit value and *f_c_* = 84 MHz. A digital counter circuit addresses each RAM location, enabling the retrieval of cosine values corresponding to the incoming signal’s sample index. The multiplication operation is gated by a control signal, ensuring synchronization by incrementing the counter only when a new sample arrives (as shown in Figure 7). The choice of a 4-bit width (1-bit for sign and 3-bit for magnitude) for the cosine samples balances the resolution requirements with the need to minimize data width expansion, resulting in a 20-bit I component after multiplication.

The quadrature-phase (Q) component is similarly generated by multiplying the input signal with a sine vector, which is stored in a Xilinx single-port RAM block as an 8k sample array of sin(2*πf_c_*), where *f_c_* = 84 MHz.

### 4.2. Optimizing Bit Width for VLSI Implementation

A previous knowledge of the data range is important so as not to reserve bit width unnecessarily. For example, if a 16-bit sample is multiplied by a 16-bit sinusoidal data point to generate an in-phase and/or a quadrature component, the bit width of the result will grow to 32 bits. Now the 32-bit sample will be multiplied by subsequent delayed samples to calculate the autocorrelation lags, which will grow the result to 64 bits. Finally, accumulating this result 10,000 times means an increase in the width by at least 14 bits, which means the final accumulated sample needs a 78-bit representation. This large width of signal representation is very expensive in terms of the FPGA circuits’ placement and wiring.

A logic design of the autocorrelation algorithm with 16-lag and full sample-width precision, i.e., down-sampling by a factor of four was attempted; however, the resulting implementation required a chip size exceeding the capacity of the available FPGA hardware. Consequently, bit-width management became a critical focus. Bit-truncation techniques were employed to reduce data width from 64-bit to 48-bit or 32-bit, depending on the number of autocorrelation lags and the down-sampling factor. In autocorrelation signal processing technique, the logic design size mainly depends on two factors: (a) the number of lags, which determines the number of real and imaginary vectors that will be calculated, accumulated, and then streamed off to the host PC and (b) down-sampling factor, which determines the size of each vector; down-sampling by a factor of four reduces the size of each vector from 8192 to 2048 samples, whereas down-sampling by a factor of two reduces the size of each vector to 4096 samples. The number of autocorrelation lags and the down-sampling factor both determine the frequency resolution and, hence, the velocity resolution as well as the maximum measurable wind velocity [11]. This creates a trade-off between the degree of bit truncation on one hand and the number of autocorrelation lags combined with the down-sampling factor on the other.

**Figure 8:**
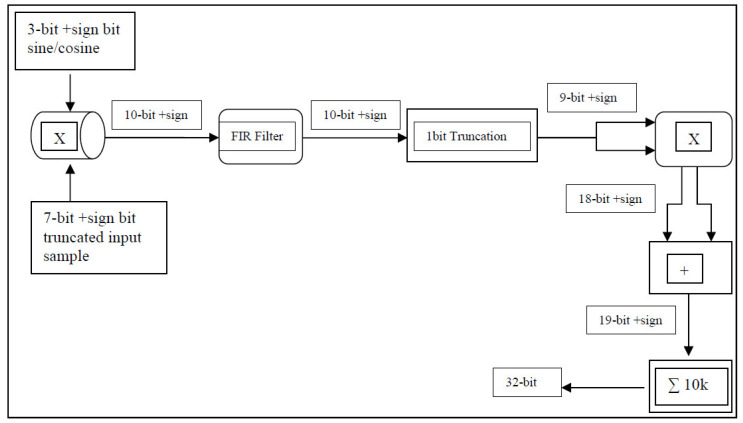
Binary data-width management in FPGA programming.

Observing the output of the optical detector while its output is set to 10× (the maximum gain) and its input is fed by both local oscillator and received signals shows that it is sampled to a range of +/−70 counts on the ADC, i.e., signed 7-bit output. The cosine and sine are set such that each has only signed 3-bit output, as shown in Figure 8. Therefore, backscattered received samples are truncated at the 8th bit. Now, the result of multiplying these received samples by the sine and cosine points produces signed 10-bit outputs. The filtered output is truncated by 1 bit then multiplied by subsequent delayed samples. Multiplying an in-phase and a quadrature sample by a subsequent delayed one involves an addition and a subtraction arithmetic operation, which causes the data width to grow from signed 9- to 18-bit and finally to signed 19-bit. Accumulating these 19-bit samples for 10k times increases their width by 14 bits, i.e., the accumulated autocorrelation lags are streamed off to the host PC as signed 32-bit points. This enabled a practical and efficient design, suitable for the FPGA while maintaining algorithmic integrity. Such an approach illustrates the importance of precise bit-width control and optimization in designing high-performance digital systems within the constraints of modern hardware.

### 4.3. Low-Pass (FIR) Filter Digital Circuit Design

**Figure 9:**
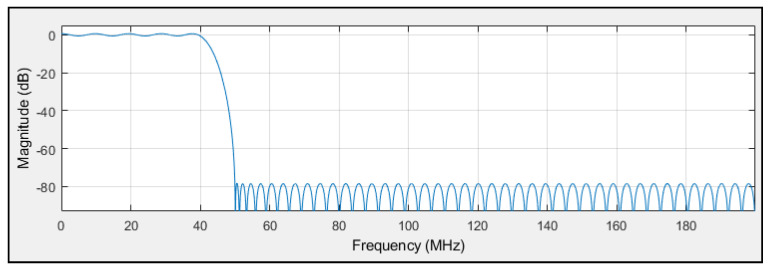
FIR low-pass filter magnitude (dB) responses.

The low-pass FIR filter logic circuit is designed to remove high-frequency components from both the in-phase (I) and quadrature (Q) signals. This functionality is implemented using a Xilinx FIR Compiler 5.0 circuit block; the filter design was developed using the Filter Design and Analysis tool in Simulink toolbox from MATLAB R2012a (version 8). The frequency response of the filter, as shown in Figure 9, demonstrates its ability to suppress out-of-band signals above 50 MHz by more than 80 dB, which ensures effective noise removal.

### 4.4. Down-Sampling Digital Circuit Design

**Figure 10:**
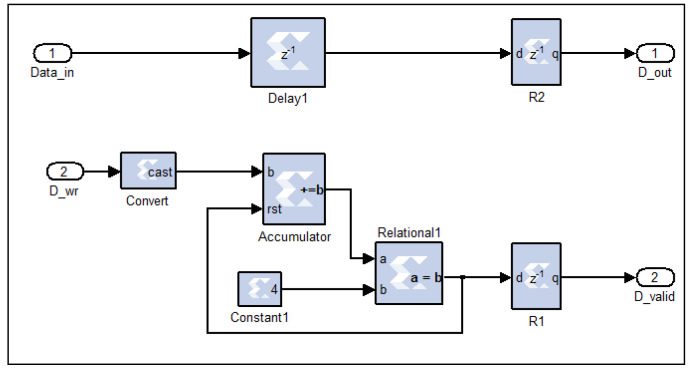
The down-converter digital circuit, as implemented on the FPGA.

This circuit performs down-conversion (decimation) of the filtered in-phase (I) and quadrature (Q) signals by a factor of four. This process involves retaining one sample for every four input samples while discarding the remaining three. The operation is achieved through a digital counter circuit, as illustrated in Figure 10, which tracks the incoming data samples by converting the Boolean-type data-enable signal associated with each sample into a fixed-unsigned type.

When the counter’s output reaches the value of four, a comparator logic circuit generates a Boolean-1 signal. This signal serves as the new data-enable control, ensuring that only the fourth sample in each group is passed through. Consequently, the valid data signal (D_valid) is reduced by a factor of four, effectively downsizing the filtered time-domain I and Q signals (Data_in) from 8k samples to 2k samples (D_out). In essence, the circuit selectively resets the data-enable signals for three samples and activates it only for the fourth, ensuring efficient data size reduction while preserving signal integrity.

### 4.5. Digital Circuit Design for Autocorrelation Computation

**Figure 11:**
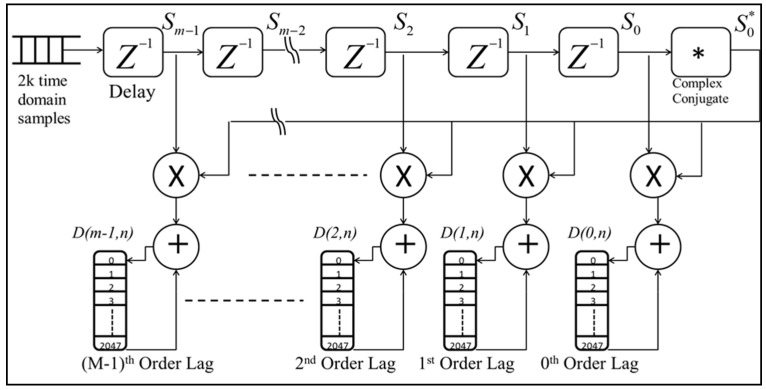
The autocorrelation circuit, as implemented on the FPGA, processes the input vector by passing it through a series of time-delay elements. This delays the signal to generate multiple lags. Simultaneously, the complex conjugate of the I-Q signal components is calculated. The conjugated signal is then multiplied with the corresponding delayed I-Q samples, producing the autocorrelation lags for each delay stage. These computed lags are subsequently passed through accumulator circuits, where data from 10,000 laser shots are aggregated. This FPGA-based design ensures efficient computation of autocorrelation lags with high precision, enabling robust signal analysis in real-time.

This circuit calculates the autocorrelation array of backscattered received signals. The input, a 2k vector of down-sampled, filtered, and digitized signals, is processed through a series of 15 delay elements, as shown in Figure 11, to compute lags ranging from 0 to 15. At each delay stage, the signal is tapped to capture the delayed samples.

After passing through the final delay element, a digital circuit converts each sample into its complex conjugate, represented as S_i_^*^ = *I_i_* − *jQ_i_*, where *Ii* and *Qi* are the in-phase and quadrature components, respectively. The complex conjugate samples are then multiplied with the corresponding delayed samples from the taps to compute *S*(*i*,*m*) = S^*^(*i*)·*S*(*i* + *m*), where *i* is the sample index and *m* is the lag number.

This process produces 16 complex vectors, each containing 2k elements. These vectors are subsequently passed to an accumulator circuit, where the autocorrelation arrays from 10,000 laser shots are aggregated for further analysis. The resulting design ensures efficient and precise computation of autocorrelation across multiple lags, facilitating robust signal processing.

**Figure 12:**
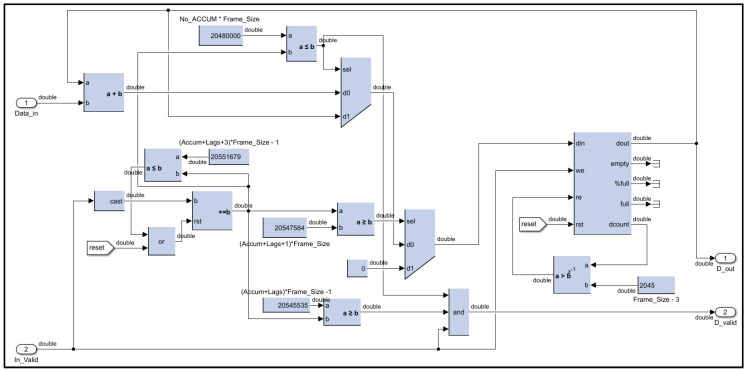
Accumulation of autocorrelation vector digital circuit design.

The accumulator circuit for the autocorrelation lags operates similarly to the accumulator in the FFT pre-processing algorithm, with added functionality tailored to its specific requirements. Once the signals from 10,000 laser shots are accumulated, additional logic ensures that both the real and imaginary components of the 12 autocorrelation lags (in the case of choosing only 12 lags) are output simultaneously. These twenty-four vectors are streamed out in order, i.e., lag-0 followed by lag-1, and so on. Therefore, all FIFOs must be reset once all vectors have been streamed out, which takes 24 additional cycles from the completion of the 10,000 pulses. In other words, FIFOs are reset every 10,024 pulses in the case of twelve lags of autocorrelation to clear the accumulated autocorrelation vectors and prepare for accumulating a new set of vectors from the next 10,000 laser shots. Further logic circuits manage the sequencing of these accumulated vectors, controlling the order in which they are streamed to the next processing stage. This design ensures synchronized data handling and efficient preparation of autocorrelation results for subsequent stages, as shown in Figure 12.

## 5. Host Computer Post-Processing and Measurements

**Figure 13:**
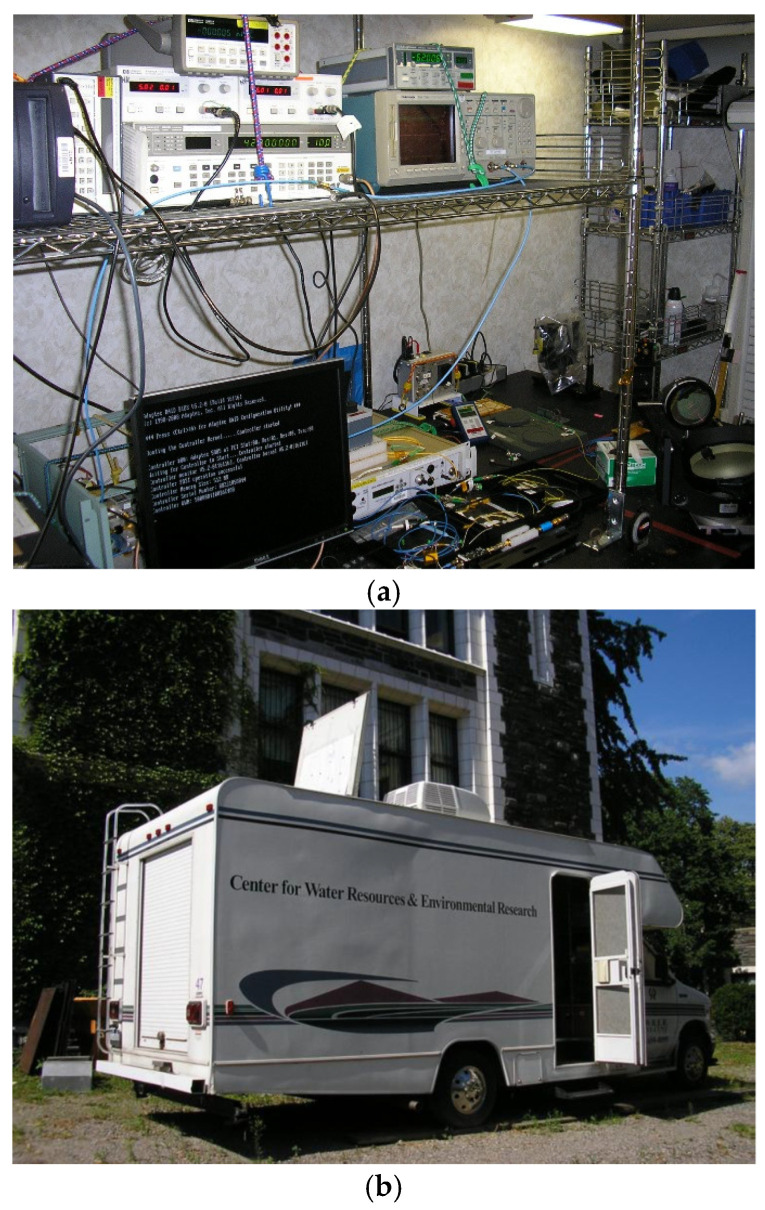
(**a**) The CDL instrument which is installed on an optical table located in our research vehicle. (**b**) The laser pulses were transmitted into the atmosphere while operating in a vertical mode through an opening in the vehicle’s roof, as shown.

After the accumulated power spectra or autocorrelation vectors are streamed from the FPGA to the host PC via the PCI Express bus, the host computer performs advanced data post-processing. This stage includes estimating critical parameters such as radial wind velocity, backscattered signal strength, and velocity statistics. The host system also manages data archiving and provides visualization of the results. The radial wind velocity *v* (m.s^−1^) is derived from the mean frequency shift ∆*f* (Hz) of the Doppler lidar signal using the following relationship:

(4) $$v=\frac{\lambda }{2}\Delta f$$

where λ (m) is the laser wavelength. As a result, the maximum radial velocity that can be measured is given by:

(5) $$v_{max}=\frac{\lambda }{2}f_{\max}$$

which is approximately 23 m/s. The frequency shift and the width of the return signal’s spectral peak are important for understanding the motion of the air particles that are being measured by a Doppler instrument. As for the spectral width, it is the main parameter for characterizing the degree of turbulence [12,13]. The Doppler frequency shift can be estimated by finding the centroid of the discrete power spectrum of the backscattered signals [13]. One easy way to find this frequency from a discrete power spectrum is to find the frequency bin with the highest power [14]. To accurately calculate the Doppler frequency shift of backscattered signals, velocity estimators were developed and investigated. Early studies on the performance of a frequency estimator formed from the peak of a discrete power spectrum for a CDL system were reported by Hardesty [13], whereas Rye and Hardesty investigated the estimation precision difference between discrete-Fourier-Transform (DFT)-based estimators and the Cramer–Rao lower bound (CRLB) [15,16]. An estimator’s precision can be determined by its variance, assuming that the probability density function of the estimator is a Gaussian, because such a distribution can be characterized by its first and second moments [16]. Frehlich and Yadlowsky presented the performance of the mean-frequency estimators in terms of the ratio of signal energy per estimate to the spectral noise level [17,18]. Wind velocity measurements are processed in real-time, thanks to FPGA pre-processing techniques that allow for streaming of either power spectrum or autocorrelation of received backscattered signals instead of raw data. The CDL instrument was installed on an optical table in our research vehicle that is located at the City College of New York (CCNY), New York, NY (latitude: 40.49° N, longitude: 73.56° W), as shown in Figure 13a,b.

**Figure 14:**
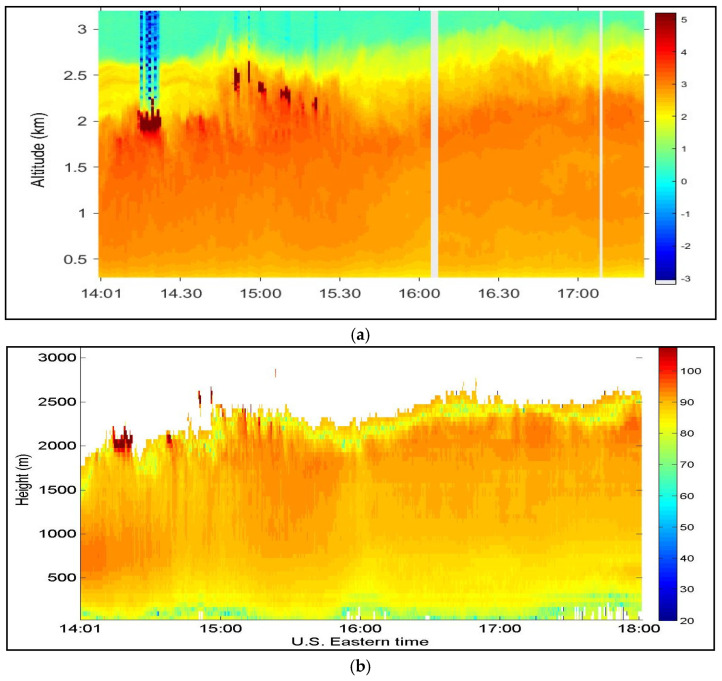
Doppler lidar’s range-corrected backscattered signals (power vs. time and height): (**a**) 1064-nm direct detection lidar and (**b**) 1.5-μm CDL measured at the Remote Sensing Laboratory of the CCNY.

To validate the data from the CDL system, a 1064-nm direct detection lidar (DDL) was also operated at the RSL of the CCNY at the same time. Figure 14a,b show the range and overlap corrected backscattered signal power of both CDL and DDL, respectively. The CDL signal power was corrected according to the inverse range dependence [19,20]. Both graphs show a strong agreement of signal intensity profiles and cloud patterns at 14:20 and 15:00. It is also clear that signal power significantly increased at a height of approximately 2000 m at 14:20 and 15:00 due to clouds at that height. Both lidars also show a gradual increase in aerosol signals as a function of time [11].

**Figure 15:**
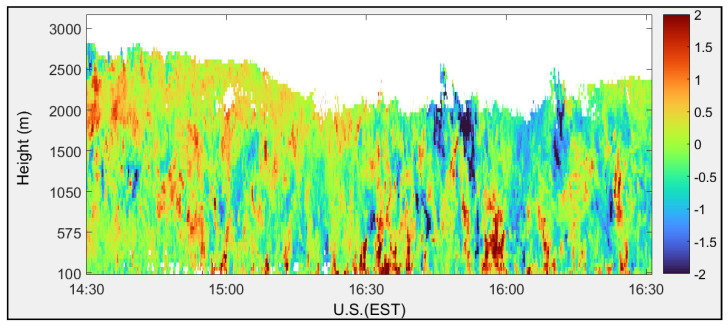
Vertical wind velocity (m/s) calculated using FFT algorithm measured at the CCNY on 17 August 2011.

**Figure 16:**
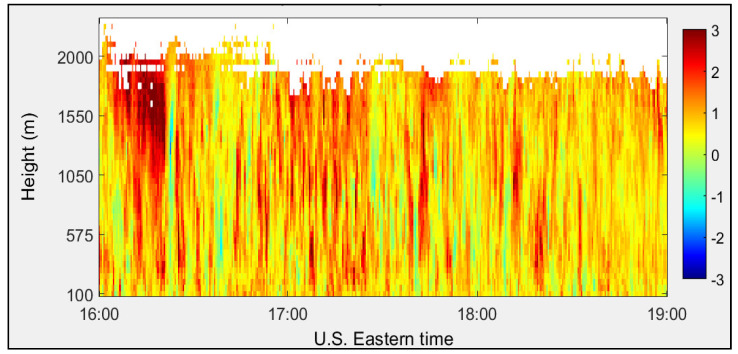
Vertical wind velocity (m/s) calculated using autocorrelation algorithm measured at the CCNY on 29 June 2012.

Vertical wind velocity profiles calculated using FFT and autocorrelation signal processing techniques are shown in Figure 15 and Figure 16, respectively. The vertical wind component displays both updrafts (red) and downdrafts (blue).

## 6. Conclusions

This study addresses the challenges and solutions in implementing FPGA-based pre-processing algorithms for estimating power spectral density and autocorrelation in a Coherent Doppler Lidar system used for wind sensing. The high data throughput of the system, coupled with the stringent timing requirements of real-time signal processing, necessitates the use of FPGA logic circuits to handle data acquisition and pre-processing efficiently.

Two primary algorithms were developed and implemented: an FFT-based method for power spectral density estimation and an autocorrelation-based method for flexible spatial resolution wind measurements. Both approaches effectively reduce data rates from the ADC to the host PC, demonstrating the FPGA’s capacity to manage high-frequency signal processing. Key design challenges such as bit growth, memory limitations, and signal drive constraints were mitigated through optimized bit-width management, custom memory architectures, and efficient logic design.

The successful implementation of these algorithms on FPGA hardware highlights its potential to meet the performance requirements of advanced Doppler Lidar systems. Additionally, the measured wind data validate the system’s capability for precise atmospheric sensing. This work underscores the critical role of hardware–software integration in addressing the computational demands of modern remote sensing applications. Future efforts may focus on further improving algorithm efficiency, exploring alternative FPGA platforms, and expanding the system’s application scope.

## Data Availability

Data are contained within the article.

## References

[1] Palacin J., Palleja T., Tresanchez M., Sanz R., Llorens J., Ribes-Dasi M., Masip J., Arno J., Escola A., Rosell J.R. (2007). Real-Time Tree-Foliage Surface Estimation Using a Ground Laser Scanner. IEEE Trans. Instrum. Meas..

[2] Huffaker R., Jelalian A., Thomson J. (1970). Laser-Doppler system for detection of aircraft trailing vortices. Proc. IEEE.

[3] Kavaya M.J., Henderson S.W., Magee J.R., Hale C.P., Huffaker R.M. (1989). Remote wind profiling with a solid-state Nd:YAG coherent lidar system. Opt. Lett..

[4] Kavaya M.J., Menzies R.T., Haner D.A., Oppenheim U.P., Flamant P.H. (1983). Target reflectance measurements for calibration of lidar atmospheric backscatter. Appl. Opt..

[5] Cariou J.P., Augere B., Valla M. (2006). Laser Source requirements for coherent lidars based on fiber technology. Comptes Rendus Phys..

[6] Karlsson C.J., Olsson F.Å., Letalick D., Harris M. (2000). All-Fiber Multifunction Continuous-Wave Coherent Laser Radar at 1.55 νm for Range, Speed, Vibration, and Wind Measurements. Appl. Opt..

[7] Abdelazim S., Santoro D., Arend M., Moshary F., Ahmed S. (2015). Development and Performance Analysis of an All-fiber Coherent Doppler Lidar System for Wind Sensing and Aerosol Profiling. IEEE Trans. Geosci. Remote Sens..

[8] Sadeghi S. (2024). Classifying FPGA Technology in Digital Signal Processing: A review. Int. J. Eng. Technol. Sci..

[9] Qi W. FPGA-based digital signal processing algorithm research. Proceedings of the 3rd International Conference on Computer Science and Information Technology.

[10] Peng X., Tang Y., Li J., Zou P., Liao P., Yan J., Shen M., Zhou L. (2024). FPGA-based CCD signal acquisition and transmission system design. Sci. Rep..

[11] Abdelazim S., Santoro D., Arend M., Moshary F., Ahmed S. (2018). A Hardware Implemented Autocorrelation Technique for Estimating Power Spectral Density for Processing Signals from a Doppler Wind Lidar System. Sensors.

[12] Zrnic D.S., Doviak R.J. (1984). Doppler Radar and Weather Observations.

[13] Hardesty R.M. (1986). Performance of a Discrete Spectral Peak Frequency Estimator for Doppler Wind Velocity Measurements. IEEE Trans. Geosci. Remote. Sens..

[14] Sirmans D., Bumgarner B. (1975). Numerical comparison of five mean frequency estimators. J. Appl. Meteorol. Climatol..

[15] Hardesty R.M., Rye B.J. (1993). Discrete spectral peak estimation in Doppler lidar. I: Incoherent accumulation and the Cramer-Rao lower bound. IEEE Trans. Geosci. Remote. Sens..

[16] Hardesty R.M., Rye B.J. (1993). Discrete spectral peak estimation in incoherent backscatter heterodyne lidar. II: Correlogram accumulation. IEEE Trans. Geosci. Remote. Sens..

[17] Frehlich R.G., Yadlowsky M.J. (1994). Performance of mean-frequency estimators for Doppler radar and lidar. J. Atmospheric Ocean. Technol..

[18] Frehlich R. (1996). Simulation of coherent Doppler lidar performance in the weak-signal regime. J. Atmospheric Ocean. Technol..

[19] Fried D. (1967). Optical heterodyne detection of an atmospherically distorted signal wave front. Proc. IEEE.

[20] Wandzura S.F., Clifford S. (1981). Monostatic heterodyne lidar performance: The effect of the turbulent atmosphere. Appl. Opt..

